# Prevention of axonal loss after immediate dosage titration of immunoglobulin in multifocal motor neuropathy

**DOI:** 10.1111/ene.16305

**Published:** 2024-04-23

**Authors:** Ali Al‐Zuhairy, Johannes Jakobsen, Christian Krarup

**Affiliations:** ^1^ Department of Neurology Copenhagen University Hospital (Rigshospitalet) Copenhagen Denmark; ^2^ Department of Clinical Neurophysiology Rigshospitalet Copenhagen Denmark; ^3^ Department of Neuroscience University of Copenhagen Copenhagen Denmark

**Keywords:** inflammatory neuropathy, multifocal motor neuropathy, neurological disorders, neuromuscular diseases

## Abstract

**Background:**

To evaluate whether ongoing axonal loss can be prevented in multifocal motor neuropathy (MMN) treated with immunoglobulin G (IgG), a group of patients with a median disease duration of 15.7 years (range: 8.3–37.8), treated with titrated dosages of immunoglobulins, was studied electrophysiologically at time of diagnosis and at follow‐up.

**Results:**

At follow‐up, the Z‐score of the compound motor action potential amplitude of the median, fibular, and tibial nerves and the neurological performances were determined. In seven patients with a treatment‐free period of 0.3 years (0.2–0.4), there was no progression of axonal loss (*p* = 0.2), whereas a trend toward further axonal loss by 1.3 Z‐scores (0.9–17.0, *p* = 0.06) was observed in five patients with a treatment‐free period of 4.0 years (0.9–9.0). The axonal loss in the group with a short treatment delay was significantly smaller than in the group with a longer treatment delay (*p* = 0.02). Also, there was an association between treatment delay and ongoing axonal loss (*p* = 0.004). The electrophysiological findings at follow‐up were associated with the isokinetic strength performance, the neurological impairment score, and the disability, supporting the clinical relevance of the electrophysiological estimate of axonal loss.

**Conclusion:**

Swift initiation of an immediately titrated IgG dosage can prevent further axonal loss and disability in continuously treated MMN patients.

## INTRODUCTION

Multifocal motor neuropathy (MMN) is characterized by slowly progressive, asymmetric weakness of the limb muscles without sensory loss [1, 2]. The electrophysiological characteristics of MMN are motor conduction block outside entrapment sites without sensory loss. In long‐term and untreated MMN, the electrophysiological findings are dominated by secondary axonal loss considered to be the main determinant of the clinical characteristics atrophy and muscular weakness [1, 2, 3, 4, 5, 6]. In MMN, it remains debated whether long‐term clinical and electrophysiological stability can be obtained in immunoglobulin G (IgG)‐treated MMN patients or whether a slow deterioration is inescapable. In two long‐term follow‐up studies of IgG‐treated MMN patients, a slight but significant deterioration of muscle performance and a progressive axonal loss were observed [7, 8]. By contrast, clinical and electrophysiological stability has been reported in another study, the conflicting results being attributed to different dosage regimens [9]. To address these discrepancies, we conducted a long‐term electrophysiological follow‐up study of treated MMN patients. We hypothesized that (i) ongoing axonal loss can be prevented in MMN provided there is swift initiation of titrated IgG therapy and (ii) the level of pretreatment axonal loss is predictive of the long‐term clinical outcome.

## METHODS

### Study population

All patients fulfilling the European Federation of Neurological Societies/Peripheral Nerve Society criteria for MMN [1] including an initial electrophysiological examination and a preserved record at the Department of Clinical Neurophysiology, Copenhagen University Hospital, diagnosed during the period from 1990 until 2014 and subsequently treated with IgG, were eligible for participation. All patients early in the disease course were given a titrated dosage of IgG (see Discussion). Exclusion criteria were other neuropathies, other neurological disorders, diabetes, and disabling musculoskeletal disorders.

### Study design

Eligible patients were examined clinically and electrophysiologically at follow‐up, the data being compared with the electrophysiological data at diagnosis. In addition, information on disease onset, course, treatment duration, and current treatment were obtained from patient records and interviews. Treatment delay occurred initially for all patients after the electrophysiological diagnosis was made. In addition, in three patients the treatment was temporarily or permanently discontinued. The total period without treatment between the diagnostic and the follow‐up electrophysiological examination was registered for all patients. The last electrophysiological examination prior to treatment initiation was applied and designated the *diagnostic* examination. The clinical and electrophysiological evaluations were separated and both examiners (A.A.‐Z. and C.K.) were mutually blinded.

The local ethics committee of the Capital Region (H‐17017657) and the Danish Data Protection Agency (2012‐58‐0004) approved the protocol. All study participants gave written informed consent.

### Clinical evaluation

Using dynamometry (Biodex Medical Systems, Shirley, NY, USA), isokinetic strength (IKS) was obtained at the wrist and ankle on the same side as examined electrophysiologically at diagnosis. To weigh all muscle groups equally, normalized strength was expressed as a percentage of the measured value compared to the predicted value, the latter being obtained from data of 178 healthy subjects previously reported [10, 11].

Neurological impairment was assessed using the Neuropathy Impairment Score (NIS) [12]. Grading of muscle strength of 21 pairs of muscle groups was scored as follows: normal strength = 0, 75% of normal strength = 1, 50% of normal strength = 2, 25% of normal strength = 3, no strength = 4. Five pairs of muscle stretch reflexes and four modalities of sensation at the index finger and hallux at both sides were scored from 0 (normal) to 2 (absent), resulting in a total maximum impairment score of 220.

The Rasch‐Built Overall Disability Scale for MMN (MMN‐RODS) [13], a self‐reporting questionnaire, was utilized for the evaluation of disability. The raw scores ranging from 0 (most severe disability) to 50 (no disability) were applied.

Finally, walking was evaluated using the Timed 25‐Foot Walk (T25FW) [14].

### Electrophysiological examination

Nerve conduction studies at follow‐up were performed in the same median, fibular, and tibial motor nerves as in the diagnostic study using near nerve needle or surface electrodes. To ascertain the long‐term effect of MMN on nerve fibers, preference was given to affected nerves that were not terminally damaged at diagnosis. In patients with both sides examined at diagnosis, the moderately affected nerves were studied at follow‐up.

Motor fibers were activated by supramaximal stimulation at the wrist and elbow in the median nerve, at the ankle and fibular head of the fibular nerve, and at the medial malleolus and popliteal fossa of the tibial nerve. As previously described, the compound motor action potentials (CMAPs) were recorded using surface electrodes in a belly‐tendon montage over the abductor pollicis brevis, extensor digitorum brevis, and abductor hallucis muscles, respectively [15, 16]. In addition, the median nerve was stimulated in the axilla and at Erb's point to detect any motor conduction block.

Due to the long interval between the two electrophysiological studies, the amplitudes of the logarithmically transformed CMAPs were compared to age‐matched normal values from our laboratory [17, 18, 19]. For each patient, a combined Z‐score for axonal loss was applied [15].

### Statistical analyses

For the primary hypothesis of an unchanged level of axonal loss during the follow‐up period, paired axonal Z‐score values were calculated as the average of the assessed nerves, including the nerves that were also examined at diagnosis. In addition, patients were divided into a group with a short total treatment‐free period of <0.5 years and a group with a long treatment‐free period between the two electrophysiological assessments of 0.5 years or longer. For the correlation between muscle strength and axonal loss, the mean normalized IKS was calculated as the average strength of the muscle groups corresponding to either of the electrophysiological examinations. The mean IKS included wrist flexion, ankle dorsal, and plantar flexion if the corresponding nerves were assessed. The correlations between axonal loss and the remaining clinical parameters were tested independent of the assessed nerves.

Descriptive data were calculated using medians and ranges. Comparisons were performed using the Wilcoxon signed rank test and the Mann–Whitney *U*‐test for paired and nonpaired comparisons, respectively. To assess correlations, the nonparametric Spearman correlation coefficient (rho) was applied. A *p*‐value of <0.05 was considered significant.

Statistics were performed using the SAS software package (SAS Institute, Cary, NC, USA).

## RESULTS

### Characteristics of the MMN patients

A total of 14 eligible patients were identified, 12 of whom (nine males) participated in the study (Table 1). The interval from symptom onset to the diagnostic electrophysiological examination was 1.7 years (range = 0.3–9.0), and the interval between the diagnostic and the follow‐up electrophysiological examination was 12.1 years (range = 4.1–32.9). The age at symptom onset was 37 years (range = 27–57) and at follow‐up 54 years (range = 42–75). The delay from symptom onset until treatment initiation was 3.4 years (range = 0.9–9.3), and the cumulated treatment‐free interval between the diagnostic and the follow‐up electrophysiological examination was 0.4 years (range = 0.2–9.0). The duration of treatment was 7.6 years (range = 3.9–28.8). During the follow‐up period, the treatment was temporarily discontinued in two patients. At time of follow‐up, 11 patients were receiving IgG at a weekly dose of 20.0 g (range = 3.7–40.0; Table 1). The diagnostic axonal Z‐score in the complete cohort was −0.6 (range= −3.8 to 1.2) and deteriorated by 0.7 (range = −0.6 to 17.0, *p* = 0.007) at follow‐up. In seven patients, a median nerve conduction block was identified at follow‐up.

**TABLE 1:** Demographics and clinical data on 12 patisents with MMN.

Variable	Median (range)
Age, years	54 (42 to 75)
Age at onset, years	37 (27 to 57)
Sex, M:F, *n*	9:3
Time since onset of MMN, years	15.7 (8.3 to 37.8)
Duration of MMN until diagnostic electrophysiological examination, years	1.7 (0.3 to 9.0)
Interval between diagnostic and follow‐up evaluations, years	12.1 (4.1 to 32.9)
Duration of MMN until treatment initiation, years	3.4 (0.9 to 9.3)
Cumulated treatment‐free interval during the follow‐up period, years	0.4 (0.2 to 9.0)
Current treatment, *n* = 11	
IgG, g/week	20.0 (3.7 to 40.0)
Isokinetic strength at follow‐up, normalized, %	66.3 (44.9 to 92.7)
NIS at follow‐up (0–220)	17.3 (0.5 to 38.5)
MMN‐RODS score at follow‐up, 0–50	47.0 (40.0 to 50.0)
Combined axonal Z‐score	
Diagnostic	−0.6 (−3.8 to 1.2)
Follow‐up	−1.5 (−15.8 to 0.2)^a^
GM1‐IgM titer, *n*	1^b^

Abbreviations: F, female; IgG, immunoglobulin G; M, male; MMN, multifocal motor neuropathy; MMN‐RODS, Rasch‐Built Overall Disability Scale for MMN; NIS, Neuropathy Impairment Score.

^a^ The follow‐up axonal Z‐score was unchanged in the seven patients with a treatment‐free interval of < 0.5 years.

^b^ One patient had both GM1 and GM2 IgM antibodies, whereas another had only GM2 IgM antibodies.

Seven patients had a short treatment‐free period between the diagnostic and the follow‐up electrophysiological examination (0.3 years, range = 0.2–0.4). In the remaining five patients, a long treatment‐free period of 4.0 years (range = 0.9–9.0) took place. In four of the five patients, this was due to a delayed treatment initiation after the diagnostic electrophysiological examination, whereas the treatment had been discontinued 8 years previously in the last patient upon his request. A slow clinical deterioration in this patient cannot be ruled out. In both groups, the treatment was temporarily discontinued for a few months in one patient. The two groups did not differ with respect to age at onset, age at follow‐up, duration of treatment, initial axonal Z‐score, or interval between the two electrophysiological examinations.

### Electrophysiological follow‐up

**FIGURE 1:**
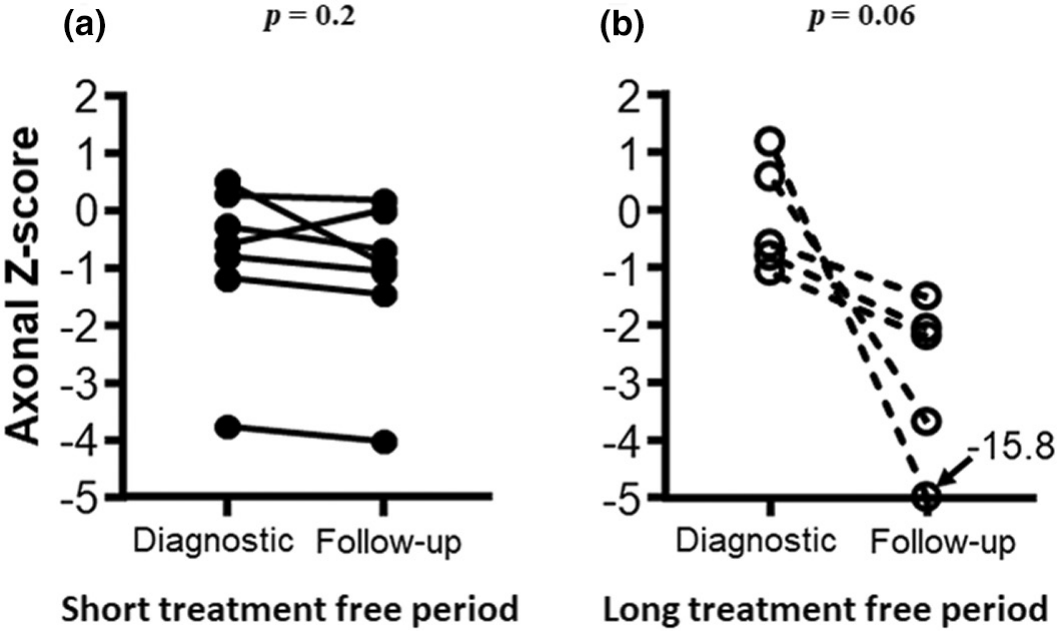
Evolution of axonal loss during 12.1 years of follow‐up. Paired values of *Z*‐scores are shown for the group with a short (<0.5 years; a) and a long (≥0.5 years) treatment‐free period (b). The axonal loss was significantly different between the two groups (*p* = 0.02).

In the seven patients with a short treatment‐free period, the diagnostic axonal Z‐score was −0.6 (range = −3.8 to 0.5) and remained unchanged at follow‐up, the difference being −0.3 (range = −1.4 to 0.6, *p* = 0.2). In the five patients with a long treatment‐free period and a diagnostic axonal Z‐score of −0.6 (range = −1.1 to 1.2), there was a trend toward a worsening by 1.3 (range = 0.9–17.0, *p* = 0.06), suggesting progressive axonal loss. In Figure 1, paired values of *Z*‐scores for the group with the short and the long treatment‐free period are shown. The axonal loss was significantly different between the two groups (*p* = 0.02). This finding is corroborated by the highly significant Spearman correlation demonstrating the relationship between the duration of the treatment‐free period and the progressive axonal loss (rho = −0.77, *p* = 0.004).

### Clinical outcome

**FIGURE 2:**
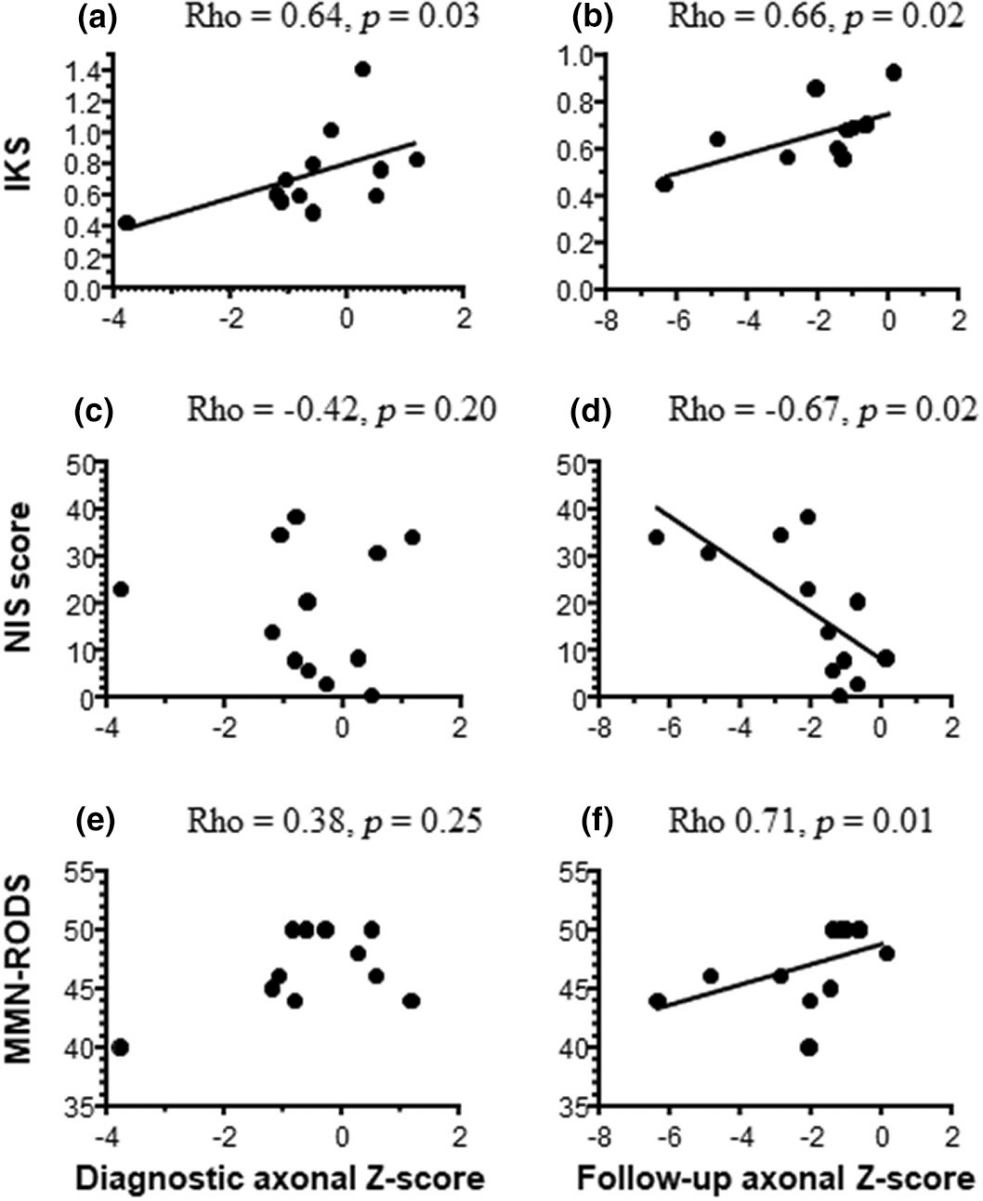
Correlations between the axonal scores and the clinical measures. The diagnostic axonal Z‐score was correlated with the isokinetic strength (IKS) at follow‐up (a), but not the Neuropathy Impairment Score (NIS; c) or the Rasch‐Built Overall Disability Scale for multifocal motor neuropathy (MMN‐RODS; e). The axonal Z‐score at follow‐up was correlated with the IKS (b), the NIS (d), and the MMN‐RODS score (f).

**FIGURE 3:**
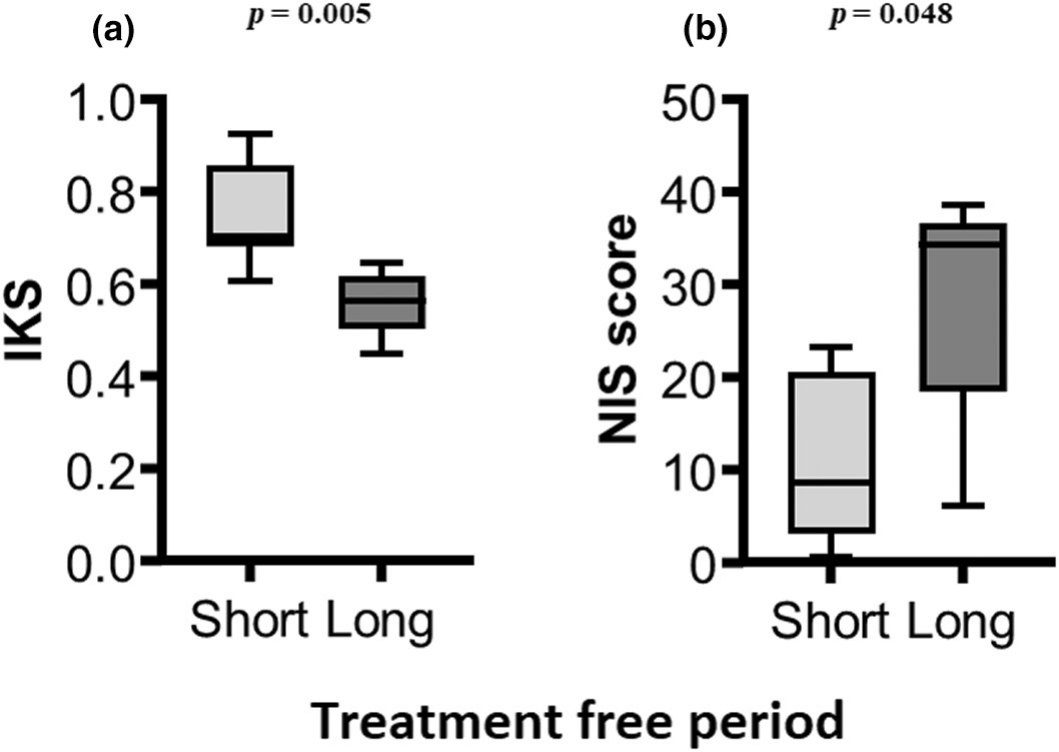
Clinical comparison of patients with short and long treatment‐free periods. Patients with a long treatment‐free period (≥0.5 years) had impaired isokinetic strength (IKS; *p* = 0.005; a) and Neuropathy Impairment Score (NIS; *p* = 0.048; b) compared to patients with a short treatment‐free period (<0.5 years).

The correlations between the axonal scores and the clinical measures are presented in Figure 2. Following adjustment for the treatment‐free period, the diagnostic axonal Z‐score was correlated with the isokinetic strength at follow‐up only (Figure 2a). Conversely, the axonal Z‐score at follow‐up was correlated with all clinical measures (Figure 2b,d,f) except the T25FW, suggesting that the finding of axonal loss is of clinical relevance. In Figure 3, it appears that patients with a long treatment‐free period had impaired IKS and NIS as compared to patients with a short treatment‐free period (*p* = 0.005 and *p* = 0.048, respectively).

## DISCUSSION

The findings of the present study suggest that in MMN further axonal damage in previously affected nerves can be prevented following continuous IgG treatment using an immediately titrated dosage regimen. Furthermore, the correlation between the axonal loss at both time points and the clinical performance at follow‐up indicates that the electrophysiological findings are of clinical relevance.

Previous clinical and electrophysiological studies have shown conflicting results as to whether continuous IgG treatment can prevent clinical and electrophysiological deterioration in the long‐term disease course. In a study from 2002 on 11 intravenous immunoglobulin (IVIg)‐treated MMN patients followed for 4–8 years, the Dutch group observed a slight but significant decrease in muscle strength at last follow‐up compared to the maximal improvement following IVIg commencement. This was accompanied by a significant reduction in the CMAP amplitude of most nerves, leading the authors to conclude that progressive axonal loss in MMN is inescapable [7]. These findings were supported by an Italian study from 2004 on 10 IVIg‐treated MMN patients followed for 5–12 years in whom a similar progressive clinical and electrophysiological deterioration took place [8].

However, a study from 2004 from the United States showed no clinical deterioration during the follow‐up period. In addition, the CMAP amplitude was unchanged, and electromyography (EMG) showed signs of ongoing axonal degeneration including fibrillation potentials and positive sharp waves in only 9% of the regions examined. In addition, there was a significant reduction in the number of regions with ongoing axonal degeneration compared to the pretreatment examination. The authors explained the observed differences by the administration of a significantly higher IgG maintenance dose as compared to the one used by the Dutch group [9].

At the last follow‐up, we observed no differences between the IgG dose applied in our cohort and the one described at the last follow‐up in the Dutch cohort (*p* = 0.1). Neither are there any obvious dose differences between the Italian study and our observations. The dose strategy during the early treatment period, however, appears different in these three studies. In the clinics in the Netherlands and Italy, an incremental dose regimen is applied, whereas a titrated dosage regimen is applied in the Danish clinics. Thus, the Dutch and the Italians apply an induction treatment with a full dose of IgG at 2 g/kg body weight (b.w.) followed by a maintenance dose of 0.4 g/kg once every 4 weeks. In the case that the neurological condition deteriorates, the treatment dose is subsequently increased [4]. Our approach is to start at the same maximum dose of IgG at 2 g/kg b.w. Then the length of the period of neurological stabilization is observed. The optimal weekly dose per kg b.w. is estimated as 2 g IgG divided by the number of weeks before the first disease symptoms reappear. Using this approach, patients typically receive twice the IVIg dose of that administered using the Dutch regimen early in the disease. In the Dutch as well as in the Italian clinics, the dose is gradually increased following clinical deterioration [4, 20, 21, 22]. This incremental IgG dose regimen leads to a gradual dose increase of approximately 50% throughout the disease course [8, 20]. In comparison, our patients showed little dose variation throughout the follow‐up period.

It is likely that the dose applied in the four studies might play a role in whether continuous axonal loss can be prevented during long‐term treatment. However, our study shows that swift initiation of IgG therapy is of importance too. In addition, the long follow‐up period studied in the present report was longer than previously reported, suggesting that the prevention of further axonal loss and disability is not time limited.

There is limited information on the association between the degree of axonal loss and disability in MMN. In the present study on MMN, the diagnostic axonal loss was predictive for isokinetic strength only. An explanation for this finding could be that weakness in MMN predominantly affects the hands, whereas most assessment scales such as NIS and MMN‐RODS encompass the neurological functions more widely. In previous reports by our group on patients with chronic inflammatory demyelinating polyneuropathy, strong associations between initial axonal loss and long‐term clinical performance scores were observed [15, 16].

The association between electrophysiological parameters and concurrent muscle strength in 39 MMN patients was evaluated in a cross‐sectional study from 2003 in which an association between a reduced distal CMAP amplitude and an increased risk of corresponding muscle weakness was observed [5]. The same group subsequently published a report in 2006 on 20 MMN patients in whom the presence of axonal loss determined by muscle EMG was the strongest indicator of corresponding muscle weakness [6]. Similar observations have been reported in 2007 [23]. In all of these cross‐sectional studies, the outcome parameter of muscle weakness was examined manually and then categorized as either present or absent, resulting in less precision. We like to emphasize that dynamometry yields continuous and precise measures of strength, thereby reducing the need for the number of patients included in clinical studies. Therefore, it is advantageous to consider the use of dynamometry during the planning in studies of small patient groups, such as MMN. Other study limitations should be considered, including the following. (i) Assessment of isokinetic strength in our study was associated with axonal loss of the corresponding nerve, but in MMN there is an irregular nerve involvement on a fascicular level [4], leading to a less relevant evaluation of strength not accurately reflecting the nerve involvement. The weakness of minor findings by the IKS score is that they do not necessarily reflect an impairment noticed by the patient or a clinically meaningful change. However, if such changes occur in conjunction with dose adjustments or a gradual worsening is observed on consecutive evaluations, then the IKS might well promote timely management in MMN. (ii) A minor fraction of the muscles involved at wrist flexion are innervated by the ulnar rather than the median nerve. (iii) The MMN‐RODS is designed to be utilized following conversion of the raw scores into Rasch‐transformed scores, as the untransformed MMN‐RODS performs suboptimally compared to the clinical findings. (iv) Determination of the CMAP amplitude as a measure of axonal loss is disputed, as both distal conduction blocks and collateral sprouting can influence the amplitude [6]. We decided to use this parameter because it gives a continuous variable and because the observed axonal loss was in accordance with our findings on EMG and motor unit number estimation. All the abovementioned limitations are of minor significance compared with the major consideration of the limited numbers of long‐term‐treated patients with this rare disorder. This questions the generalization of our findings but does not take away the immediate challenge of what is a sufficient treatment dose of IgG in MMN.

In conclusion, the present study indicates (i) that ongoing axonal loss in MMN might well be avoided using immediate dosage titration of IgG and (ii) that delay of IgG treatment leads to permanent axonal loss and muscle weakness.

## AUTHOR CONTRIBUTIONS

**Ali Al‐Zuhairy:** Conceptualization; investigation; writing – original draft; methodology; formal analysis; project administration; writing – review and editing; visualization. **Johannes Jakobsen:** Conceptualization; funding acquisition; writing – review and editing; supervision; methodology. **Christian Krarup:** Conceptualization; investigation; methodology; writing – review and editing; supervision; visualization.

## FUNDING INFORMATION

This study was funded by Rigshospitalet and by an Investigator‐Initiated Research Grant (No. IISR‐2019‐104354) from Takeda Pharma, a company incorporated in Denmark.

## CONFLICT OF INTEREST STATEMENT

A.A.‐Z. and C.K. have no conflict of interest. J.J. received an Investigator‐Initiated Research Grant (No. IISR‐2019‐104354) from Takeda Pharma.

## Data Availability

The data that support the findings of this study are available from the corresponding author upon reasonable request.
